# Coronavirus disease – 2019 assessment zone: A community hospital's rapid response to a novel infectious pandemic

**DOI:** 10.1017/cem.2020.391

**Published:** 2020-05-08

**Authors:** Rohit Mohindra, Cori Atlin, Carla Moran, Ann Shook, Andrea Ennis, Jennifer Page, Marisa Vaglica, Paul Hannam

**Affiliations:** North York General Hospital, Emergency Department, Toronto, Ontario

**Keywords:** COVID-19, emergency medicine, infectious disease, quality improvement

Dear Editor,

During the coronavirus disease–2019 (COVID-19) pandemic, emergency departments (EDs) have had to adapt to assessing higher volumes of patients under droplet isolation. Hospital crowding remains a challenge at baseline in Ontario.^1^ The enhanced protection zone was created out of the necessity for physical space in which to safely assess patients. Here we briefly describe our enhanced protection zone with the hope that others may benefit from our experience.

**Figure 1.**
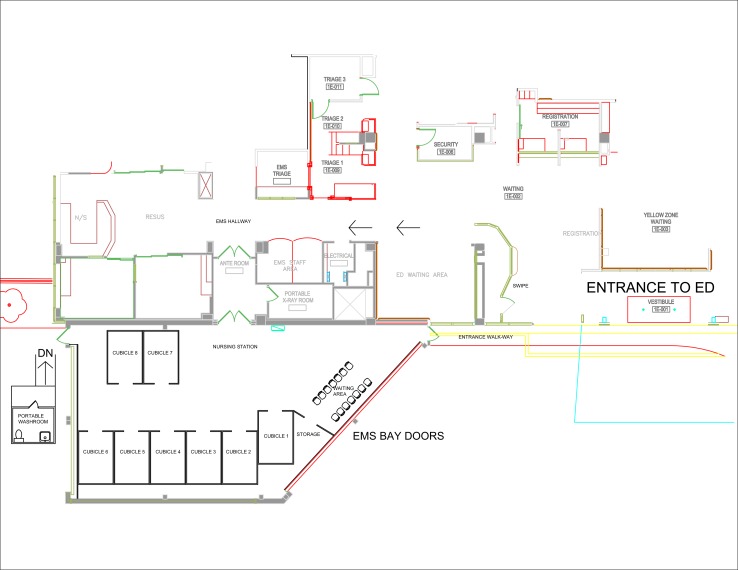
Blueprint for the enhanced protection zone.

North York General Hospital (NYGH) is a large-sized academic community hospital, situated in Toronto, Ontario, serving a catchment area of 420,000 people. Our annual ED consensus was 113,976 in 2018–2019. In 2003, NYGH was directly affected by severe acute respiratory syndrome (SARS) and built an assessment zone using our ambulance bay.^2^ A similar approach has been undertaken now. Our clinical coordinator adapted the existing blueprints (Figure 1), mapped out the physical space, and sought in situ feedback from frontline staff. It was constructed over 33 hours and fully functional 30 minutes after opening.

The enhanced protection zone is situated in the ambulance bay. It has a separate entrance and waiting room and is equipped with heating, ventilation, electricity, and an external portable washroom. The walls are made of poly sheathing, which can be cleaned with bleach. The zone has three mobile computers and capacity for bloodwork, electrocardiogram, and portable X-ray. There are eight assessment rooms; nursing and physician tables; and space for support staff, including clerical and facility. Each room has its own stretcher, stethoscope, and donning and doffing area.

The average daily ED census in March 2018 and 2019 were 309 and 315, respectively. The day the enhanced protection zone opened, the ED census was 477. During the first 9 days that the enhanced protection zone was open, the ED received an average of 441 patients per day, with 44% being assessed in the enhanced protection zone. To meet the needs of our diverse patients, we developed translated discharge instructions in different languages, including simplified Chinese and Farsi. Additionally, we created a website for patients to access their COVID-19 swab results.

The enhanced protection zone represents our solution to assessing significantly higher volumes of patients while adhering to infection prevention standards. It was constructed in less than 1.5 days. Once alternative COVID-19 assessment clinics have opened, the enhanced protection zone will have the capacity to house stretcher-bound patients who are not critically ill, leaving the ED for sicker patients. This design represents a viable model for others who need a rapidly deployable physical area, separate from the main ED.
